# Assessment of Changes in Serum C-Reactive Protein Levels in Patients after Ischemic Stroke Undergoing Rehabilitation—A Retrospective Observational Study

**DOI:** 10.3390/jcm12031029

**Published:** 2023-01-29

**Authors:** Wojciech Borowicz, Kuba Ptaszkowski, Lucyna Ptaszkowska, Eugenia Murawska-Ciałowicz, Joanna Rosińczuk

**Affiliations:** 1 Department of Pediatric Infectious Diseases, Wroclaw Medical University, 50-368 Wroclaw, Poland; 2 Department of Nursing and Obstetrics, Wroclaw Medical University, 51-618 Wroclaw, Poland; 3 Department of Physiotherapy, Wroclaw Medical University, 50-368 Wroclaw, Poland; 4 Institute of Health Science, University of Opole, Katowicka 68, 45-060 Opole, Poland; 5 Physiology and Biochemistry Department, Wroclaw University of Health and Sport Sciences, 51-612 Wroclaw, Poland

**Keywords:** stroke, biomarkers, C-reactive protein, functional outcomes, rehabilitation

## Abstract

Inflammation plays a key role in the pathogenesis and prognosis of ischemic stroke. C-reactive protein (CRP) is an inflammatory biomarker of inflammation and may reflect the progression of vascular disease. Using a biomarker such as CRP to predict recovery rather than mortality may present clinical value in providing rehabilitation. The primary aim of the study was to analyze changes in serum CRP levels in patients after ischemic stroke during the regenerative-compensatory period and to assess the usefulness of CRP as a potential biomarker during the rehabilitation period. The project was carried out as a retrospective analysis of prospectively collected data from post-stroke patients from the Department of Neurological Rehabilitation of the Regional Specialist Hospital in Wrocław. A group of 52 patients, after their first-ever ischemic stroke with subsequent hemiplegia, was finally qualified to participate in the study. Serum CRP levels were determined during blood laboratory tests. The Modified Rankin Scale (mRS) and Barthel Index (BI) were used to assess functional outcomes. Rehabilitation using neurophysiological methods was applied five days a week (each session lasted 60 min, and the entire period was 42 days). At the first test, serum CRP levels were found to be above 5 mg/L in 19 patients, the second test in 12 patients, the third test in five patients, and the fourth test in 9 patients. Only three patients had values higher than 5 mg/L in all consecutive assessments (*p* > 0.05). There was a statistically significant increase in BI scores after therapy (*p* < 0.001) as well as a decrease in the mRS score by 2.2 points (*p* < 0.001), in CRP values by 5.02 mg/L (*p* = 0.019), and in cortisol levels by 2.5 nmol/L (*p* = 0.002). Statistically significant relationships were observed between the CRP levels after rehabilitation and the corresponding mRS scores (rs = 0.29, *p* = 0.038). Furthermore, the effect of BMI on CRP levels was demonstrated (B = 0.20, *p* = 0.038). In conclusion, despite demonstrating a significant relationship between CRP levels and corresponding mRS scores, CRP levels alone may not serve as an independent predictor of long-term functional outcomes in ischemic stroke patients undergoing rehabilitation.

## 1. Introduction

Stroke is the third leading cause of death in the adult population, following heart disease and cancer. Post-stroke mortality rates in Poland are higher than in other European countries and the USA. Long-term disability is a serious problem among survivors. Studies have shown that almost 15–30% of people are permanently disabled after a stroke, with more than 20% of those requiring institutional support three months after a stroke [1]. Given the high burden of disability after stroke, there is a need to identify clinical biomarkers so that individualized treatment regimens can be developed after ischemic stroke and targeted to maximize function and quality of life [2]. Inflammation plays a key role in the pathogenesis and prognosis of ischemic stroke [3].

C-reactive protein (CRP) is an inflammatory biomarker of inflammation and may reflect the progression of vascular disease. The entire production process of this protein takes place in response to pro-inflammatory cytokines and other inflammatory mediators. Conflicting evidence implies that CRP may be a prognostic biomarker of ischemic stroke outcome. As reported in the literature, most studies that analyzed the relationship between CRP and ischemic stroke outcomes used mortality or subsequent vascular events as the primary outcome measure. However, given that almost half of the post-stroke patients experience moderate to severe functional impairment, using a biomarker such as CRP to predict recovery rather than mortality may present clinical value during rehabilitation [4].

CRP enhances nerve cell damage by increasing the activation of the complement system. There is a proven close relationship between the increase in CRP levels and complement components in the blood of patients in the acute phase of stroke and the size of the infarct focus. The progression or enlargement of the zones of damage to brain tissue following stroke is a consequence of the deterioration of local microcirculatory conditions. In addition, a cascade of destructive phenomena associated with inflammatory and immunological reactions in response to ischemic damage to neural tissue is observed. It is important to approach this problem in the context of the development of stroke focal damages and the potential possibilities of modulating the inflammatory response in acute focal cerebral ischemia. Therefore, more effective treatment methods for patients in the acute phase of stroke and post-stroke rehabilitation will be developed [5].

The primary aim of the study was to analyze factors affecting serum CRP levels in patients after ischemic stroke during the regenerative-compensatory period. The secondary aim was to assess the usefulness of CRP as a potential biomarker during rehabilitation, including the relationship between CRP levels and the functional assessment of post-stroke patients.

## 2. Materials and Methods

### 2.1. Ethical Consideration

The study was approved by the Bioethics Committee of the Wrocław Medical University (KB–813/2020) and conducted in accordance with the Good Clinical Practice Guidelines and the Declaration of Helsinki. All project participants were informed of the purpose of the study and how it would be conducted, and gave their written consent to participation in the study and the processing of their personal data. Moreover, the study was registered under the number ISRCTN16891871.

### 2.2. Participants and Design

The project was carried out as a retrospective analysis of prospectively collected data from 62 patients from the Department of Neurological Rehabilitation of the Regional Specialist Hospital in Wrocław. The study included a group of 62 first-ever ischemic stroke survivors discharged from neurology or internal medicine wards with a recommendation for rehabilitation no later than 14 days after discharge from the stroke unit. All patients were Caucasian and were rehabilitated from 2 January 2021 to 22 December 2021. The patients were treated for mobility issues for 42 days. Patients were qualified for the project by an interdisciplinary team consisting of a neurologist, a specialist in medical rehabilitation, and a physiotherapist. The selection of patients to participate in the study was purposive.

### 2.3. Qualification Procedure

The eligibility criteria for all participants included first-ever ischemic stroke confirmed by MRI or CT, subsequent hemiplegia, functional disability assessed on the day of admission on the modified Rankin Scale (mRS) > 3, consent to participate in the study, consent from the attending physician, the absence of infection, which was defined by excluding patients with fever, signs of infection on physical examination, or those who needed antibiotic therapy during subsequent hospitalization. Furthermore, all patients underwent a routine chest X-ray, urinalysis, and consultation with a specialist in internal medicine within the first two days of admission. Furthermore, a follow-up internist consultation was conducted when CRP values increased above 10 mg/L before discharge from the ward. Exclusion criteria comprised patients with sensory aphasia identified by anamnesis and medical examination, a history of myocardial infarction, chronic respiratory disease (bronchial asthma, COPD), previously diagnosed persistent musculoskeletal dysfunction, active infection (chest X-ray, general urinalysis, full results of physical examination), and patients who did not give their consent to participate. 

A group of 52 patients who met the inclusion criteria was finally qualified to participate in the study. The group included 18 women (aged 67.00 ± 8.70) and 34 men (aged 62.00 ± 9.00). The patients underwent neurological rehabilitation for six weeks. At two-week intervals, the patients had their blood drawn for routine tests, and selected biochemical parameters were determined in the remaining blood. All tests and surveys were carried out by the same physician.

### 2.4. Outcome Measures

Patients had their blood drawn from a basilic vein every fortnight, i.e., on admission to the ward, and after two, four, and six weeks of therapy. Blood for the laboratory tests was always taken at the same time, i.e., at 6:30 a.m. and on an empty stomach. The researchers kept in mind that the CRP and leukocyte levels could also be affected by measures taken under different clinical conditions, such as different times of day or after consuming food. To minimize errors and eliminate confounding factors, all patients in our study always had their blood drawn at the same time (6:30 a.m.), and had not taken non-steroidal anti-inflammatory drugs.

Furthermore, CRP levels were determined in the blood. Serum CRP assessments were performed using the Alinity c CRP Vario assay. It is an immunochemical test that uses latex particles to measure serum and plasma CRP levels in an accurate and precise manner. When an antigen-antibody reaction occurs between the CRP protein present in the test sample and an antibody against the CRP protein adsorbed on latex particles, the agglutination process takes place. The agglutination process is detected as a change in absorbance (572 nm), with the rate of change being proportional to the amount of CRP present in the sample [6].

Patients’ functional outcomes were assessed using the modified Rankin Scale (mRS) [7,8] and Barthel Index (BI) [9,10], the most commonly used clinimetric tools for measuring disability after stroke. Long-term functional outcome scales, such as mRS and BI, are commonly used for measuring the degree of physical dependence and have high inter-rater reliability compared to other scales [11]. Therefore, both scales were used in the project. BI was performed on the day of admission to the ward and after 21 days of rehabilitation and after 42 days of treatment for mobility issues. In contrast, mRS was performed only on the day of admission to the ward and after six weeks. Functional disability was defined as an mRS score > 3. This study assessed the relationship between BI scores and CRP values at three-time points after ischemic stroke in the regenerative-compensatory period, and the relationship between mRS and CRP levels at two-time points.

### 2.5. Neurological Rehabilitation

After stabilizing the patient’s condition during the early neurological rehabilitation period, rehabilitation using neurophysiological methods was applied five days a week in patients whose dysfunctions had not diminished. Each session of neurorehabilitation lasted 60 min, and the entire period was 42 days. Blood pressure and heart rate were measured before each kinesitherapy unit for patient safety reasons. Treatment for mobility issues has always been selected individually for each person, taking into account the patient’s capabilities.

### 2.6. Statistical Analysis

The statistical analysis was performed using Statistica 13.1 software (TIBCO, Inc., Palo Alto, CA, USA). The sample size was assessed based on the available results in the unit’s database (*n* = 12). Means and standard deviations of CRP results (before and after rehabilitation) were used in the analysis of estimating the sample size. The estimated sample size was calculated by a paired-means test (paired *t*-test). The alpha level was set at 0.05, and the power of the test at 0.8. It also assumed no correlation of evaluated variables and adopted a two-sided null hypothesis. On the basis of the parameters, the estimated sample size was obtained for 49 patients.

Arithmetic means, standard deviations, and the range of variability (extreme values) were calculated for measurable variables. Prevalence (%) was calculated for qualitative variables. All studied quantitative variables were verified using the Shapiro-Wilk test to determine distribution type. Comparisons between pre- and post-therapy results were made using the *t*-test for independent samples. Moreover, a Pearson correlation analysis was performed between the selected variables. In addition, an analysis of the effect of selected factors on the BI score was performed using linear regression. The next step was to build a multivariate model. The model-building process was conducted using progressive stepwise regression. The level of α = 0.05 was used for all comparisons.

## 3. Results

Table 1 shows the characteristics of the group, including age, height, weight, BMI, number of days since stroke diagnosis, NIHSS score, sex, smoking, and history of chronic disease.

Table 2 shows the results of CRP tests in each measurement. The results were not statistically significantly different (*p* > 0.05). At the first test, serum CRP levels were found to be above 5 mg/L in 19 patients, the second test in 12 patients, the third test in five patients, and the fourth test in nine patients. Only three patients had values higher than 5 mg/L in all consecutive assessments. 

Table 3 shows a comparison of results before and after rehabilitation. There was a statistically significant increase in BI scores after therapy (*p* < 0.001). Moreover, there was a statistically significant decrease in the mRS score by 2.2 points (*p* < 0.001), in CRP values by 5.02 mg/L (*p* = 0.019), and in cortisol levels by an average of 2.5 nmol/L (*p* = 0.002).

Furthermore, a correlation analysis of the results of CRP levels and other variables was also performed, as shown in Table 4. Statistically significant relationships were observed between the results of CRP levels after rehabilitation and the corresponding mRS scores (r_s_ = 0.29, *p* = 0.038) plus platelet (PLT) values (r_s_ = 0.30, *p* = 0.036). 

The effect of the selected parameters on final CRP levels (a univariate model of the predictors included in the analysis) was also assessed after the completion of the treatment for mobility issues. The unstandardized and standardized regression coefficients, standard error, and level of statistical significance were determined. The following variables were included in the analysis: age, sex, BMI, smoking, history of diabetes, hypertension, mRS (before and after rehabilitation) and BI (before and after) scores, and blood biochemical parameters (before and after). 

Table 5 shows the linear regression analysis in the univariate model. The effect of BMI (B = 0.20, *p* = 0.038) on CRP levels was demonstrated. The linear regression analysis in a multivariate model (stepwise, progressive) revealed an effect of BMI (B = 0.26, *p* = 0.005), cortisol value (B = 0.28, *p* = 0.023) and mRS score (B = 2.43, *p* = 0.005) on CRP levels. Moreover, Table S1 presents univariate logistic regression analyses assessing the effect of selected variables on CRP score (≤5 vs. >5). Table S2 presents multivariate logistic regression analyses assessing the effect of selected variables on CRP scores (≤5 vs. >5).

## 4. Discussion

Comprehensive rehabilitation is a fundamental component of therapeutic management, through which patients achieve functional improvement and independence. In addition, rehabilitation provided by an interdisciplinary team forms a key element in reducing the risk of death, severe disability, and the stress associated with the need to adapt to life under conditions altered by the disease.

According to the authors, the novelty of this study is the in-depth assessment of CRP levels in first-ever ischemic stroke patients in the regenerative-compensatory period undergoing neurological rehabilitation as part of a clinical trial based on a strict protocol of inclusion and exclusion criteria, using both objective measurements and subjective scales recommended in the literature, with a uniform statistical design.

According to the available literature, there have been numerous attempts to assess both CRP levels in post-stroke patients in the acute phase and the impact of stroke on the functional outcome of this group of patients, whereas there are few papers on the correlation of CRP levels and its effect on the functional outcome of patients after ischemic stroke in the regenerative-compensatory period.

Wnuk et al. [12] observed that non-infectious CRP values are an independent risk factor for poor short- and long-term functional outcomes with ischemic stroke patients undergoing thrombolytic treatment. Those authors found that a poor functional outcome, as assessed by mRS >3, was achieved by patients with CRP levels >8.65 mg/L compared to those with CRP levels below 5 mg/L. In our project, we assessed a similar to the above-mentioned report relationship between CRP levels on admission and functional outcome assessed by mRS on the admission of patients to the ward and a follow-up measurement after 42 days of the treatment for mobility issues. We defined functional disability as a mRS score >3; the group size was smaller, but the age range was comparable. We observed statistical relationships between the results of CRP levels after 42 days of rehabilitation and the corresponding mRS scores (r_s_ = 0.29, *p* = 0.038) plus platelet (PLT) values (r_s_ = 0.30, *p* = 0. 036), which may reflect the complex interactions of the induction and progression processes occurring between vascular wall cells (endothelial cells, tunica media myocytes) and blood cells (leukocytes and platelets) plus plasma lipoproteins.

As highlighted by Masztalewicz et al. [13], the contribution of inflammatory and immunological factors to ischemic stroke is mainly considered in terms of their effect on the development of atherosclerosis and the destabilization of atherosclerotic plaques within the arteries supplying the brain. As the literature supports the view that elevated leukocyte values may also be a predictor of poorer functional outcomes, the authors of this project attempted to verify this finding. These authors [13] emphasize the important role of inflammatory mechanisms in the dynamics of stroke focus development. An acute inflammatory response develops in response to the necrotic tissues, or, more specifically, to the antigens released from them, which contributes to the enlargement of the infarcted area as reflected in the neurological deterioration, and significantly affects the subsequent prognosis of the stroke patient. Microglia cells, astrocytes, T lymphocytes, endothelial cells, perivascular cells (macrophages), and neurons located in the ischemic area are involved in this reaction. Peripheral blood leukocytes (neutrophils, monocytes, T lymphocytes), flowing into the vicinity of the developing lesions are also involved in this reaction. The influx of leukocytes is determined by the reperfusion of the cerebral vessels in the ischemic area. The increased adhesion of these cells to the endothelial surface impairs local blood flow. Again, there is a reduction in the oxygen and glucose supply to the brain area affected by stroke and, consequently, a worsening of the damage associated with ischemia. Animal experiments have proven that counteracting leukocyte adhesion to the endothelium results in beneficial therapeutic effects in animals in the form of a reduction in brain infarct volume and associated edema and a reduction in a neurological deficit. The migration of leukocytes into the central nervous system (CNS) exacerbates inflammation-related damage to ischemic brain tissues, as these cells are a source of free radical substances, proteolytic enzymes, neurotoxic cytokines, and neurotoxic nitric oxide [13]. In this study, we found no significant statistical relationship between elevated white blood cell counts and poorer functional outcomes as assessed by BI and mRS.

To date, there is no universally accepted cut-off point in the literature for the infectious CRP levels in studies on ischemic stroke [14]. Although a specific cut-off value (>6 mg/L) was used to rule out a possible comorbid infection in the study by Montaner et al. [15], they emphasize that clinical assessment plays a more important role in ruling out infection. Therefore, previous studies may have produced inconsistent results regarding the prognostic role of CRP in ischemic stroke patients due to the variable and inadequate CRP threshold. It should be borne in mind that CRP and leukocyte values may also be affected by measurements that took place under different clinical conditions, such as a different time of day, after food intake and under the influence of non-steroidal anti-inflammatory drugs. For example, fasting is associated with a significant decrease in CRP levels, as highlighted by Alam et al. [16] in their study, whereas leukocyte counts increase by almost 10% two hours after eating a meal [12].

In our study, to minimize errors, all patients had their blood drawn at the same time (6.30 a.m.) and were not taking non-steroidal anti-inflammatory drugs. This was our way of trying to eliminate confounding factors. For example, high blood pressure (hypertension) is a major global problem; it is the second risk factor after age that can lead to stroke [17].

Peng et al. [18] found in their studies that increased CRP levels tended to be a more significant risk factor for stroke among women than men, which they confirmed by the multivariate logistic analysis. Chinese researchers obtained different results and found that elevated CRP levels significantly affected male patients but not female patients [19]. The inconsistent findings on sex differences may have been partly due to genetic and hormonal differences between men and women. The above-mentioned processes need to be clarified; consequently, further research is needed to work out the phenomenon. In this study, due to the small group size, there was no breakdown by sex.

Rajeshwar et al. [20] revealed that higher levels of hs-CRP were significantly associated with poor outcomes after considering several confounding variables. Similarly, a study by Winbeck et al. [21] revealed that CRP levels within 12 to 24 h at the onset of stroke symptoms were independently predictive of adverse functional outcomes after one year of follow-up. CRP levels measured 24 to 48 h after the onset of symptoms were even stronger predictors; however, the timeframe did not meet the inclusion criteria, as it falls outside the time window for assessing acute inflammation.

Elevated CRP levels revealed positively significant associations with long-term, >30 days, adverse functional outcomes. Song et al. [22] found that CRP levels were significantly and positively correlated with functional outcomes, assessed by mRS after 1, 3, 6, and 12 months of follow-up, with increasingly strong observational associations as time increased. A significant highlight was that CRP values measured seven days after admission showed a stronger statistical correlation with mRS scores after 12 months than CRP values measured within 24 h of admission.

The detailed mechanism of reduced functional outcome after ischemic stroke is not yet fully understood and is related to a complex cycle of interconnected molecular and cellular mechanisms. However, some studies by, among others, Jayaraj et al. [23], point out that inflammation in the surviving phase after stroke can promote tissue repair and functional regeneration.

Ye et al. [24] found elevated CRP values as an independent predictor of functional disability after one year in both sexes, in men (*p* = 0.017) and in women (*p* = 0.042). In contrast, Ahmadi Ahangar et al. [25] found that serum CRP values were positive in 122 cases (57%). Out of 122 cases of positive CRP, 64 cases (52%) involved women, and the remaining 58 cases (48%) involved men (*p* = 0.21). The results of this study revealed that positive serum CRP values were associated with ischemic stroke severity and poor prognosis. In contrast, Totan et al. [17] examined the correlation between CRP values and the degree of motor deficit in mRS. Motor deficits were prevalent in the study group, with CRP values in the range of 5–50 mg/dL. There were no significant differences in the cases studied between patients with severe disabilities and patients with moderate disabilities.

The effectiveness and need for the patients’ mobilization are evidenced by the obtained functional outcomes of the study group of patients before and after rehabilitation (shown in Table 4). This study revealed a statistically significant increase in BI scores after therapy (*p* < 0.001). Moreover, there was a statistically significant decrease in the mRS score by 2.2 points (*p* < 0.001), in CRP values by 5.02 mg/L (*p* = 0.019), and in cortisol levels by an average of 2.5 nmol/L (*p* = 0.002).

In this study, we used a multivariate analysis to determine whether CRP values were an independent predictor of long-term functional outcomes while controlling for confounding variables. The linear regression analysis in a univariate model revealed an effect of BMI (B = 0.20, *p* = 0.038) on CRP values. The linear regression analysis in a multivariate model (stepwise, progressive) revealed an effect of BMI (B = 0.26, *p* = 0.005), cortisol level (B = 0.28, *p* = 0.023) and mRS score (B = 2.43, *p* = 0.005) on CRP values.

Chinese researchers Gu Hong-Qiu et al. [3] found that less than 20% of poor functional outcomes could be explained by recurrent stroke, meaning that more than 80% of functional damage is due to disability. Therefore, typical secondary prevention strategies to prevent stroke recurrence, which include rehabilitation, are very important. Pawluk et al. [26] pointed out that the precise and reliable process of reduced functional outcome after ischemic stroke is not yet fully understood and is related to a complex cycle of interconnected molecular and cellular mechanisms. As they point out, inflammation contributes to cell death, brain damage, and blood-brain barrier disruption. Furthermore, inflammation plays a role in pathogenesis and progression, increases the risk of stroke, and only later causes functional disability. In contrast, Jayaraj et al. [23] emphasized that chronic inflammation could promote tissue repair and functional regeneration in the chronic phase after stroke.

The results presented above provide a lot of interesting and relevant information, which has cognitive and practical relevance for rehabilitation planning in patients after ischemic stroke in the regenerative-compensatory period. However, further research is required to implement this knowledge in clinical practice.

### Study Limitations

A limitation of this study was that our results might also be biased by unidentified confounding factors that were not adapted. In addition, our results concerned only patients from one facility and cannot be generalized to a larger population. Therefore, the relationship between plasma CRP values and functional outcomes after ischemic stroke during the regenerative-compensatory period should be further validated in other cohorts.

Long-term functional outcome rating scales, such as BI and mRS, are commonly used for measuring functional outcome assessment and have high inter-item reliability. There is no mandatory definition of good and unfavorable outcomes using BI and mRS. The subjective standards determining the outcome are set by the researcher. It should be borne in mind that psychological conditions such as post-stroke anxiety and depression are psychological effects that can affect functioning. Altered mental status and comorbid depression are known to affect the quality of life; however, they are not measured with these clinimetric tools. Altered mental status and comorbid depression are important clinical problems associated with stroke, and some studies report a correlation between altered mental status after stroke and elevated CRP. A more holistic assessment of outcomes using scales that take into account both physical and psychological well-being may better characterize the overall disability after stroke in terms of long-term outcomes

## 5. Conclusions

This study showed that despite demonstrating a significant relationship between CRP levels and corresponding mRS scores, CRP levels alone may not serve as an independent predictor of long-term functional outcomes in ischemic stroke patients undergoing rehabilitation. CRP levels in ischemic stroke patients in the regenerative-compensatory period are affected by BMI, cortisol levels (stress hormone), and disability score assessed by mRS. The clinical utility of CRP levels in the rehabilitation of post-ischemic stroke patients in the regenerative-compensatory period during the treatment for mobility issues should be further validated based on multicenter trials regarding the care of post-ischemic stroke patients in the regenerative-compensatory period during the treatment for mobility issues.

## Figures and Tables

**Table 1:** Characteristics of the study group.

Variable	Study Group (*n* = 52)			
	$\bar{x}$	SD	Min	Max
Age [years]	65.8	9.33	48.0	83.0
Height [cm]	167.86	8.67	150.00	186.00
Body weight [kg]	73.74	14.40	46.00	108.00
BMI [kg/m^2^]	26.12	4.21	16.33	35.44
Time since stroke onset [days]	23.9	2.76	18.0	30.0
NIHSS [score]	17.1	1.08	16.0	19.0
Sex	F—*n* = 18; 32.7% M—*n* = 34; 67.3%			
Hypertension	No—*n* = 13; 24.0% Yes—*n* = 39; 76.0%			
Diabetes	No—*n* = 33; 64.0% Yes—*n* = 19; 36.0%			
Smoking	No—*n* = 32; 62.0% Yes—*n* = 20; 38.0%			

Abbreviations: *n*, number of participants; $\bar{x}$, mean; SD, standard deviation; Min, minimum value; Max, maximum value; F, Female; M, Male; BMI, body mass index, NIHSS, National Institutes of Health Stroke Scale.

**Table 2:** Numerical characteristics of the results of individual CRP sampling.

Sample Number	*n*	Min–Max [mg/L]	$\bar{x}\;[\mathrm{mg}/L]$	SD [mg/L]
Admission Test 1	52	0.4–70.3	8.1	14.6
After 2 weeks Test 2	52	0.4–31.9	4.3	5.88
After 4 weeks Test 3	52	0.4–26.7	3.0	4.09
After 6 weeks Test 4	52	0.4–12.7	3.1	2.88
*p*-value	0.94 *			

Abbreviations: *n*, number of participants; $\bar{x}$, mean; SD, standard deviation; Min, minimum value; Max, maximum value. * one-way ANOVA.

**Table 3:** Comparisons of results before and after rehabilitation.

	Before				After				Difference				*p*-Value
	$\bar{x}$	SD	Min	Max	$\bar{x}$	SD	Min	Max	$\bar{x}$	SD	Min	Max	
BI	9.6	1.58	7.0	13.0	16.62	1.12	14.0	19.0	7.02	1.19	5.0	10.0	<0.001
mRS	3.48	0.5	3.0	4.0	1.28	0.45	1.0	2.0	−2.2	0.45	−3.0	−1.0	<0.001
CRP [mg/L]	8.08	14.59	0.4	70.3	3.06	2.88	0.40	12.7	−5.02	13.97	−63.9	8.7	0.019
COR [nmol/L]	15.92	4.7	6.4	29.7	13.42	3.13	7.20	20.6	−2.5	3.85	−11.3	6.0	0.002
Hemoglobin [g/dL]	13.93	1.64	10.7	17.2	13.99	1.37	10.6	16.0	0.06	1.09	−2.5	3.3	0.835
RBC [T/L]	4.48	0.51	3.45	5.98	4.6	0.49	3.57	5.56	0.13	0.34	−0.5	0.9	0.208
WBC [thous./µL]	7.39	2.3	3.96	14.05	6.95	1.91	4.42	14.2	−0.44	2.22	−7.1	3.9	0.296
PLT [thous./µL]	242.48	67.42	103.0	472.0	260.14	67.72	119.0	410.0	17.66	59.76	−136.0	173.0	0.194
HCT [%]	40.6	4.14	30.7	50.3	40.79	3.83	32.40	49.1	0.19	2.41	−6.7	6.2	0.816

Abbreviations: *n*, number of participants; $\bar{x}$, mean; SD, standard deviation; Min, minimum value; Max, maximum value; BI, Barthel Index; mRS, modified Rankin Scale; CRP, C-reactive protein; COR, cortisol; RBC, red blood cell; WBC, white blood cell; PLT, plates; HCT, hematocrit. All significant values are marked in bold.

**Table 4:** Correlations between CRP levels and other variables.

			mRS	BI	COR	HB	RBC	WBC	PLT	HTC
CRP	Before treatment	coefficient r	0.18	−0.13	0.16	−0.26	−0.18	0.13	−0.01	−0.20
		*p*-value	0.221	0.372	0.261	0.066	0.202	0.369	0.946	0.170
	After treatment	coefficient r	0.29	−0.06	0.07	−0.09	0.12	0.13	0.30	−0.01
		*p*-value	0.038	0.686	0.640	0.514	0.412	0.384	0.036	0.943
	Difference	coefficient r	0.19	−0.04	0.08	0.00	0.06	0.20	0.07	0.07
		*p*-value	0.194	0.800	0.577	0.995	0.688	0.164	0.612	0.627

Abbreviations: CRP, C-reactive protein; BI, Barthel Index; mRS, modified Rankin Scale; COR, cortisol; RBC, red blood cell; WBC, white blood cell; PLT, plates; HCT, hematocrit.

**Table 5:** Linear regression analyses assessing the effect of selected variables on CRP score.

		Univariate Linear Regression Analysis					Multivariate Linear Regression Analysis				
		B	SE	t	*p*-Value	ß	B	SE	t	*p*-Value	ß
Age		0.00	0.05	−0.06	0.95	−0.01	-	-	-	-	-
BMI *		0.20	0.09	2.14	0.038	0.29	0.26	0.09	2.94	0.005	0.38
Time since stroke onset		0.07	0.73	0.10	0.920	0.02	-	-	-	-	-
NIHSS		2.29	1.84	1.25	0.22	0.18	-	-	-	-	-
Sex	M	Ref.					-				
	F	−0.11	0.45	−0.24	0.81	−0.04	-	-	-	-	-
Hypertension	No	Ref.					-				
	Yes *	−0.78	0.47	−1.66	0.10	−0.23	-	-	-	-	-
Diabetes	No	Ref.					-				
	Yes	0.11	0.43	0.27	0.79	0.04	-	-	-	-	-
Smoking	No	Ref.					-				
	Yes	−0.06	0.42	−0.13	0.90	−0.02	-	-	-	-	-
mRS–before		0.64	0.82	0.78	0.44	0.11	-	-	-	-	-
mRS–after *		1.61	0.89	1.81	0.08	0.25	2.43	0.83	2.94	0.005	0.38
BI–before		−0.23	0.26	−0.86	0.39	−0.12	-	-	-	-	-
BI–after		−0.02	0.37	−0.05	0.96	−0.01	-	-	-	-	-
COR [nmol/L]–before		0.07	0.09	0.80	0.43	0.11	-	-	-	-	-
COR [nmol/L]–after *		0.21	0.13	1.62	0.11	0.23	0.28	0.12	2.36	0.023	0.30
HGB [g/dL] *–before		−0.25	0.25	−1.00	0.32	−0.14	-	-	-	-	-
HGB [g/dL] *–after		−0.07	0.30	−0.24	0.81	−0.03	-	-	-	-	-
RBC [T/L]–before		−0.25	0.82	−0.30	0.76	−0.04	-	-	-	-	-
RBC [T/L]–after *		0.90	0.85	1.06	0.29	0.15	-	-	-	-	-
WBC [thous./µL]–before		0.30	0.18	1.71	0.09	0.24	-	-	-	-	-
WBC [thous./µL]–after *		0.38	0.21	1.81	0.08	0.25	-	-	-	-	-
PLT [thous./µL]–before		0.00	0.01	−0.17	0.86	−0.02	-	-	-	-	-
PLT [thous./µL]–after *		0.01	0.01	2.07	0.054	0.29	-	-	-	-	-
HTC [%]–before		0.00	0.10	−0.01	0.99	0.00	-	-	-	-	-
HTC [%]–after		0.04	0.11	0.38	0.71	0.05	-	-	-	-	-

Abbreviations: BMI, body mass index, NIHSS, National Institutes of Health Stroke Scale; M, Male; F, Female; BI, Barthel Index; mRS, modified Rankin Scale; COR, cortisol; HGB, hemoglobin, RBC, red blood cell; WBC, white blood cell; PLT, plates; HCT, hematocrit. *Notes:* B—unstandardized regression coefficient B; SE—standard error; t: B/standard error; ß—standardized regression coefficient ß; * variables included in a multivariate model (criterion: *p* < 0.3 in a univariate model).

## Data Availability

The data presented in this study are available on request from the corresponding author.

## Supplementary Materials

The following supporting information can be downloaded at: https://www.mdpi.com/article/10.3390/jcm12031029/s1, Table S1: Univariate logistic regression analyses assessing the effect of selected variables on CRP score (≤5 vs. >5). Table S2. Multivariate logistic regression analyses assessing the effect of selected variables on CRP score (≤5 vs. >5).
