# Supplementary material for: Assessment of Changes in Serum C-Reactive Protein Levels in Patients after Ischemic Stroke Undergoing Rehabilitation—A Retrospective Observational Study
Source: J Clin Med. 2023 Jan 29;12(3):1029. doi: 10.3390/jcm12031029 (PMC9917915; doi:10.3390/jcm12031029)
Supplement: Supplementary file 1 [file jcm-12-01029-s001.zip › jcm-2142686-supplementary.pdf]

Table S1. Univariate logistic regression analyses assessing the effect of selected variables on CRP score (≤5 vs. >5).

		Univariate logistic regression analysis									
		CRP>5 before					CRP>5 after				
		B	p-value	OR	CI95%		B	p-value	OR	CI95%	
**Age**		0.03	0.44	1.03	0.96	1.09	0.02	0.58	1.02	0.94	1.11
**BMI**		0.03	0.70	1.03	0.90	1.18	0.20	0.05	1.23	1.00	1.50
**Time since stroke onset**		0.07	0.50	1.07	0.87	1.32	0.16	0.23	1.17	0.90	1.52
**NIHSS**		-0.24	0.39	0.79	0.45	1.37	-0.19	0.60	0.83	0.41	1.67
**Sex**	M	Ref.					Ref.				
	F	-0.48	0.45	0.62	0.17	2.18	-0.63	0.47	0.53	0.10	2.91
**Hypertension**	No	Ref.					Ref.				
	Yes	-1.11	0.10	0.33	0.09	1.25	-1.19	0.12	0.30	0.07	1.39
**Diabetes**	No	Ref.					Ref.				
	Yes	0.79	0.19	2.20	0.67	7.22	-0.14	0.85	0.87	0.19	3.98
**Smoking**	No	Ref.					Ref.				
	Yes	-0.08	0.89	0.92	0.28	3.00	-0.25	0.75	0.78	0.17	3.58
**mRS**		0.64	0.28	1.90	0.60	6.05	0.91	0.23	2.48	0.56	11.07
**BI**		-0.01	0.94	0.99	0.68	1.42	-0.29	0.40	0.75	0.39	1.46
**COR [nmol/l]**		0.10	0.12	1.11	0.97	1.26	0.29	0.03	1.33	1.03	1.72
**HGB [g/dl]**		-0.35	0.08	0.71	0.48	1.04	-0.06	0.81	0.94	0.56	1.58
**RBC [T/l]**		-1.04	0.13	0.35	0.09	1.34	0.68	0.40	1.97	0.41	9.47
**WBC [thous./µl]**		0.07	0.60	1.07	0.83	1.37	0.20	0.26	1.22	0.86	1.72
**PLT [thous./µl]**		0.00	0.60	1.00	0.99	1.01	0.01	0.27	1.01	1.00	1.02
**HTC [%]**		-0.11	0.15	0.89	0.77	1.04	0.04	0.71	1.04	0.86	1.26

Abbreviations: BMI, body mass index; NIHSS, National Institutes of Health Stroke Scale; M, Male; F, Female; BI, Barthel Index; mRS, modified Rankin Scale; COR, cortisol; HGB, hemoglobin; RBC, red blood cell; WBC, white blood cell; PLT, plates; HCT, hematocrit. *Notes:* B – unstandardized regression coefficient B; OR – odds ratio; CI – confidence intervals.

Table S2. Multivariate logistic regression analyses assessing the effect of selected variables on CRP score (≤5 vs. >5).

		Multivariate logistic regression analysis									
		CRP>5 before					CRP>5 after				
		B	p-value	OR	CI95%		B	p-value	OR	CI95%	
**Age**		-	-	-	-	-	-	-	-	-	-
**BMI**		-	-	-	-	-	0.061	0.448	1.062	0.909	1.242
**Time since stroke onset**		-	-	-	-	-	0.053	0.660	1.054	0.833	1.334
**NIHSS**		-	-	-	-	-	-	-	-	-	-
**Sex**	M			-					-		
	F	-	-	-	-	-	-	-	-	-	-
**Hypertension**	No			-					-		
	Yes	-0.785	0.365	0.456	0.084	-0.720	0.347	0.347	0.487	0.109	2.180
**Diabetes**	No			-					-		
	Yes	0.963	0.181	2.618	0.640	10.710	-	-	-	-	-
**Smoking**	No			-					Ref.		
	Yes	-	-	-	-	-	-	-	-	-	-
**mRS**		0.401	0.562	1.493	0.386	5.771	1.435	0.071	4.199	0.885	19.926
**BI**		-	-	-	-	-	-	-	-	-	-
**COR [nmol/l]**		0.078	0.305	1.081	0.931	1.256	0.198	0.093	1.219	0.967	1.535
**HGB [g/dl]**		-0.298	0.554	0.742	0.276	1.995	-	-	-	-	-
**RBC [T/l]**		-1.300	0.364	0.272	0.016	4.517	-	-	-	-	-
**WBC [thous./µl]**		-	-	-	-	-	0.005	0.979	1.005	0.682	1.483
**PLT [thous./µl]**		-	-	-	-	-	0.006	0.309	1.006	0.994	1.018
**HTC [%]**		0.130	0.589	1.139	0.710	1.829	-	-	-	-	-

Abbreviations: BMI, body mass index; NIHSS, National Institutes of Health Stroke Scale; M, Male; F, Female; BI, Barthel Index; mRS, modified Rankin Scale; COR, cortisol; HGB, hemoglobin; RBC, red blood cell; WBC, white blood cell; PLT, plates; HCT, hematocrit. *Notes:* B – unstandardized regression coefficient B; OR – odds ratio; CI – confidence intervals.
